# Effect of Age on the Initiation of Biologic Agent Therapy in Patients With Inflammatory Bowel Disease: Korean Common Data Model Cohort Study

**DOI:** 10.2196/15124

**Published:** 2020-04-15

**Authors:** Youn I Choi, Yoon Jae Kim, Jun-Won Chung, Kyoung Oh Kim, Hakki Kim, Rae Woong Park, Dong Kyun Park

**Affiliations:** 1 Department of Gastroenterology Gil Medical Center Gachon University College of Internal Medicine Incheon Republic of Korea; 2 Health IT Research Center Gil Medical Center Gachon University Incheon Republic of Korea; 3 Ajou Medical Center Suwon Republic of Korea

**Keywords:** ulcerative colitis, Crohn’s disease, early-onset, late-onset, common data model

## Abstract

**Background:**

The Observational Health Data Sciences and Informatics (OHDSI) network is an international collaboration established to apply open-source data analytics to a large network of health databases, including the Korean common data model (K-CDM) network.

**Objective:**

The aim of this study is to analyze the effect that age at diagnosis has on the prognosis of inflammatory bowel disease (IBD) in Korea using a CDM network database.

**Methods:**

We retrospectively analyzed the K-CDM network database from 2005 to 2015. We transformed the electronic medical record into the CDM version 5.0 used in OHDSI. A worsened IBD prognosis was defined as the initiation of therapy with biologic agents, including infliximab and adalimumab. To evaluate the effect that age at diagnosis had on the prognosis of IBD, we divided the patients into an early-onset (EO) IBD group (age at diagnosis <40 years) and a late-onset (LO) IBD group (age at diagnosis ≥40 years) with the cutoff value of age at diagnosis as 40 years, which was calculated using the Youden index method. We then used the logrank test and Cox proportional hazards model to analyze the effect that age at diagnosis (EO group vs LO group) had on the prognosis in patients with IBD.

**Results:**

A total of 3480 patients were enrolled. There was 2017 patients with ulcerative colitis (UC) and 1463 with Crohn’s disease (CD). The median follow up period was 109.5 weeks. The EO UC group was statistically significant and showed less event-free survival (ie, experiences of biologic agents) than the LO UC group (*P*<.001). In CD, the EO CD group showed less event-free survival (ie, experiences of biologic agents) than the LO CD group. In the Cox proportional hazard analysis, the odds ratio (OR) of the EO UC group on experiences of biologic agents compared with the LO UC group was 2.3 (95% CI 1.3-3.8, *P*=.002). The OR of the EO CD group on experiences of biologic agents compared with the LO CD group was 5.4 (95% CI 1.9-14.9, *P*=.001).

**Conclusions:**

The EO IBD group showed a worse prognosis than the LO IBD group in Korean patients with IBD. In addition, this study successfully verified the CDM model in gastrointestinal research.

## Introduction

The incidence of inflammatory bowel disease (IBD) is increasing in newly industrialized and westernized countries [1-5]. Although the incidence of IBD in western countries is stabilizing, its prevalence remains less than 0.3%. A major issue among IBD patients is the deterioration in disease-related events [1,6,7].

Effective management of IBD requires the ability to predict and prevent acute exacerbation events [1], and several studies have focused on prognostic factors for IBD [5,8-11]. Dulai et al [12] in the United States demonstrated that a history of biologic agent use, bowel surgery, fistulizing events, baseline albumin levels, and C-reactive protein levels are associated with the prognosis of Crohn's disease (CD). Khan et al [13] reported that early corticosteroid use is an independent risk factor for the prognosis of ulcerative colitis (UC). Baars et al [14] showed that late-onset (LO) IBD is associated with the development of colorectal cancer, and Israeli et al [15] reported that early-onset (EO) IBD is associated with worse outcomes, more complex diseases, and the need for surgery.

However, data regarding the factors associated with a poor prognosis of IBD are inconclusive, particularly for the second exacerbation event after diagnosis of IBD. Moreover, there is little data available related to the prediction of IBD prognosis, especially in Asian patients.

To identify factors at the time of diagnosis that are associated with the prognosis of IBD, we used the verified Korean common data model (K-CDM) network [16,17]. The K-CDM, which follows the policy of the Observational Health Data Sciences and Informatics (OHDSI) network [18,19], is an electronic medical record (EMR) standard. The CDM has evolved since its launch in the latter half of 2016. The network facilitates the performance of efficient and transparent multicenter studies [16,17]. However, the K-CDM has not been applied to gastrointestinal research.

This study was performed to evaluate the effect of age at diagnosis on the prognosis of IBD by using the CDM format of OHDSI resources, and to assess the effectiveness of a new methodology that codes algorithms via K-CDM of OHDSI network.

## Methods

### Institutional Ethic Review Board Approval of the Study Design

The Institutional Review Board of Gil Medical Center (GMC) reviewed the study protocol (certification number: GAIRB2018-127). Since the data were analyzed anonymously, consent was not obtained.

### Financial Support

This research was supported by the Basic Science Research Program through the National Research Foundation of Korea, funded by the Ministry of Education (2017R1D1A1B03034546), and supported by a grant from the Korea Health Technology R&D Project through the Korea Health Industry Development Institute (KHIDI), funded by the Ministry of Health & Welfare, Republic of Korea (grant number: HI14C3201).

### The OHDSI Network and Korean Common Data Model Resources

The OHDSI network is an international collaboration that aims to develop data-sharing systems [18,19] by applying open-source data analytics to a large number of health databases. Each member of the OHDSI network transfers their EMR databases to the CDM.

The K-CDM is based on the OHDSI database framework (CDM version 5.0). The OHDSI network launched in 2015, and the K-CDM launched in the latter half of 2016. The uploading of the EMRs from Korean hospitals into the K-CDM continued until the second half of 2019. More detailed information regarding the extract, transform, load system of longitudinal health care databases into the CDM has been described in previous studies [20-22].

### Study Design and Data Sources

We conducted a multicenter, retrospective, cross-sectional study of the clinical history, medical treatment history, and laboratory parameters of patients with IBD according to their age at diagnosis of IBD using the K-CDM network resources.

To assess the effectiveness of our methodology, we used the CDM coding algorithms. The tertiary centers in the K-CDM use the same EMRs; therefore, we queried their CDM databases to extract the data of interest [20-22].

### Identification of Patients With Inflammatory Bowel Disease

The K-CDM database was used to identify all patients diagnosed for the first time with UC or CD (according to the International Classification of Disease codes) from January 1, 2006, to December 31, 2016.

**Figure 1:**
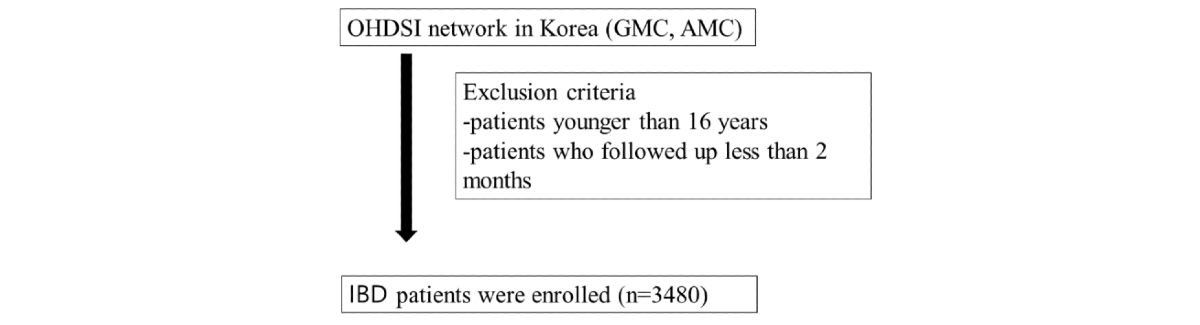
Study flow. OHDSI: Observational Health Data Sciences and Informatics Network; GMC: Gil Medical Center; AMC: Ajou Medical Center; IBD: inflammatory bowel disease.

We included patients who were followed up with for at least 2 months and excluded those misdiagnosed with other chronic IBDs including intestinal tuberculosis [23,24]. Tuberculosis is endemic in Korea, and thus intestinal tuberculosis is not rare [25]. To prevent misdiagnosis of intestinal tuberculosis as IBD or vice versa, a 2‑month course of anti-tuberculosis agents and a follow-up colonoscopy are recommended [23] (Figure 1).

### Definitions of Early-Onset and Late-Onset Inflammatory Bowel Disease

EO and LO IBD were defined as patients being diagnosed younger than 40 years and 40 years of age or older, respectively. To avoid the misclassification of LO IBD caused by loss of medical records, we designed a washout period of 1 year. Since IBD disease is a chronic and life-long disorder, using a 1 year washout period prevents misconduct in this study.

### Outcome Measures

A worsened prognosis of IBD was defined as initiation of biologic-agent therapy. Unlike other nations, in Korea, physicians are not allowed to prescribe biologic agents to patients with IBD who are diagnosed as IBD for the first time, even with severe disease activity. Biologic agents are only prescribed for patients with IBD who are unresponsive to, dependent on, or contraindicated for steroids or immunosuppressants [26-32]. Therefore, biologic-agent therapy is typically delayed until the second exacerbation event or until the patient is unresponsive to or dependent on steroids or immunosuppressants for at least 3 months after the diagnosis of IBD. In Korea, use of biologic agents is indicative of a poor prognosis [28,32-34].

### Variables

We assessed the following variables: date of the initial diagnosis of IBD, age at initial diagnosis of IBD, current age, sex, laboratory parameters, and history of IBD treatment (including systemic steroids and immunosuppressants). Treatment history was extracted from the CDM databases of the participating institutions. We regarded use of systemic steroids or immunosuppressants at diagnosis as indicators of disease activity at diagnosis. The Korean IBD treatment guidelines state that systemic steroids or immunosuppressive agents should be used only in patients with moderate or severe diseases [33,35].

### Statistical Analysis

Since there have been debates on whether age at diagnosis is independent of risk factors for worsening prognosis in IBD patients, we investigated the effect of age at diagnosis on the prognosis of IBD patients using the OHDSI K-CDM network database.

We calculated the cutoff value of age at diagnosis to predict a worsened prognosis (use of biologic agents) in IBD patients from the GMC registry using the Youden index method. Using this process, we determined the cutoff values of age at diagnosis (<40 years of age and ≥40 years of age), which showed the best performance of prognosis prediction for patients with IBD.

We then externally validated whether the cutoff values of age at diagnosis (<40 years vs ≥40 years) showed a reasonable prediction of a worsened prognosis in patients with IBD using the K-CDM network database.

The cumulative incidence (Kaplan–Meier method) of using biologic agents throughout the follow-up period according to age group was evaluated by the logrank test. The hazard ratio for the initiation of biologic agents was compared between patients with EO vs LO UC and patients with EO vs LO CD. All statistical tests were two-sided, and a value of *P*<.05 was considered indicative of statistical significance. The data was analyzed using SPSS Statistics version 22 (IBM, Armonk, NY) and MedCalc version 12.2.1 (MedCalc Software, Ostend, Belgium).

## Results

### Clinical Characteristics and Outcomes

From 2005 to 2015, 3480 patients were diagnosed with incident IBD, of whom 2017 (57.96%) had UC and 1463 (42.04%) had CD (Table 1). The median follow-up duration from the date of initial diagnosis of IBD was 109.5 weeks. The mean ages at diagnosis of EO UC (1015, 50.32%) and LO UC (1002, 49.68%) were 25.7 and 55.4 years, respectively. The mean ages at diagnosis of EO CD (1059, 72.39%) and LO CD (404, 27.61%) were 21.9 and 55.0 years, respectively.

**Table 1:** Baseline characteristics of all patients with inflammatory bowel disease (N=3480).

Characteristics			Ulcerative colitis (N=2017)	Crohn’s disease (N=1463)
Follow-up period (weeks), mean (range)			132.73 (26.43-318.92)	87.43 (14.01-248.42)
Male, n (%)			1153 (57.16)	939 (64.18)
**Age of participants**				
	Current age (years), mean (SD)		49.91 (16.92)	48.94 (18.43)
	**Age at diagnosis (years), mean (SD)**		**41.40 (17.61)**	**29.72 (17.10)**
		Age at diagnosis <40, n (%)	1015 (50.32)	1059 (72.39)
		Age at diagnosis ≥40, n (%)	1002 (49.68)	404 (27.61)
**Phenotype of IBD^a^**				
	Systemic steroid use at diagnosis, n (%)		261 (12.94)	183 (12.51)
**IBD related outcome (biologic agent)**				
	**Age at IBD related event, mean (SD)**		**39.51 (16.39)**	**31.40 (14.81)**
		Experience of biologic agent, n (%)	104 (5.16)	177 (12.10)
**Laboratory data (at diagnosis)**				
	Hematocrit (%), mean (SD)		38.89 (5.59)	38.38 (5.63)
	Serum total bilirubin (mg/dL), mean (SD)		0.71 (0.52)	0.59 (0.38)
	Serum albumin (g/dL), mean (SD)		4.12 (0.51)	3.99 (0.61)
	Serum creatinine (mg/dL), mean (SD)		1.12 (4.82)	0.81 (0.72)
	Serum C-reactive protein (g/dL), mean (SD)		1.81 (3.69)	2.39 (3.98)

^a^IBD: inflammatory bowel disease.

### Association Between Age at Diagnosis and Ulcerative Colitis or Crohn's Disease Phenotype

The rate of previous use of systemic steroid therapy at the time of diagnosis was not significantly different in the EO UC group than in the LO UC group (131/1015, 12.91% vs 130/1002,12.97%, *P*=.91) (Table 2); however, the rate was significantly higher in the EO CD group than in the LO CD group (144/1059, 13.60% vs 39/404, 9.7%, *P*=.04) (Table 3).

Previous biologic-agent therapy, serum albumin, and blood urea nitrogen differed significantly between the EO UC and LO UC groups (Table 2).

Systemic steroid use at diagnosis, previous biologic-agent therapy, male sex, age, hematocrit levels, serum total bilirubin, and serum creatinine levels differed significantly between the EO CD and LO CD groups (Table 3).

**Table 2:** Univariate analysis biologic agent experience between early onset and late onset groups in ulcerative colitis (N=2017).

Characteristics		Early onset UC^a^ (age at diagnosis <40 years) (N=1015)	Late onset UC (age at diagnosis ≥40 years) (N=1002)	*P* value
Follow-up period (weeks), mean (SD)		156.90 (156.70)	190.70 (175.80)	.005
Male, n (%)		590 (58.13)	563 (56.19)	.40
Current age (years), mean (SD)		33.60 (10.00)	63.90 (11.10)	<.001
**Phenotype of IBD^b^**				
	Systemic steroid use at diagnosis, n (%)	131 (12.91)	130 (12.97)	.91
**IBD related outcome (biologic agent)**				
	Age at experience of biologic agent (years), mean (SD)	26.13 (8.81)	54.93(9.42)	<.001
	Experienced biologic agent, n (%)	64 (6.31)	40 (3.99)	<.001
**Laboratory data (at diagnosis)**				
	Hematocrit (%), mean (SD)	39.21 (5.99)	38.71 (5.22)	.32
	Serum albumin (g/dL), mean (SD)	4.19 (0.59)	4.12 (0.51)	<.001
	Serum blood urea nitrogen (mg/dL), mean (SD)	11.09 (3.68)	14.39 (5.48)	<.001
	Serum creatinine (mg/dL), mean (SD)	0.83 (0.42)	1.29 (6.49)	.23
	C-reactive protein (g/dL), mean (SD)	2.01 (3.71)	1.69 (3.59)	.32

^a^UC: ulcerative colitis.

^b^IBD: inflammatory bowel disease.

**Table 3:** Univariate Analysis of biologic agent experience between early onset and late onset group in Crohn's disease (N=1463).

Characteristics		Early onset CD^a^ (age at diagnosis <40 years) (N=1059)	Late onset CD (age at diagnosis ≥40 years) (N=404)	*P* value
Follow-up period (weeks), mean (SD)		106.29 (125.91)	163.48 (155.93)	<.001
Male, n (%)		728 (68.74)	211 (52.23)	<.001
Current age (years), mean (SD)		28.81 (9.69)	63.32 (12.11)	<.001
**Phenotype of IBD^b^**				
	Systemic steroid use at diagnosis, n (%)	144 (13.60)	39 (9.65)	.04
**IBD related outcome (biologic agent)**				
	Age at experience of biologic agent (years), mean (SD)	23.48 (8.53)	54.09 (10.32)	<.001
	Experience of biologic agent, n (%)	144 (13.60)	33 (8.17)	<.001
**Laboratory data (at diagnosis)**				
	Hematocrit (%), mean (SD)	38.91 (5.18)	36.72 (6.34)	.001
	Serum total bilirubin (mg/dL), mean (SD)	0.62 (0.39)	0.74 (0.42)	.04
	Serum albumin (g/dL), mean (SD)	4.11 (0.57)	4.02 (0.63)	.31
	Serum creatinine (mg/dL), mean (SD)	0.73 (0.42)	1.14 (1.27)	.007
	C-reactive protein (g/dL), mean (SD)	3.69 (4.01)	2.2 (3.99)	.59

^a^CD: Crohn’s disease.

^b^IBD: inflammatory bowel disease.

### Association Between Age at Diagnosis and Initiation of Biologic-Agent Therapy

**Figure 2:**
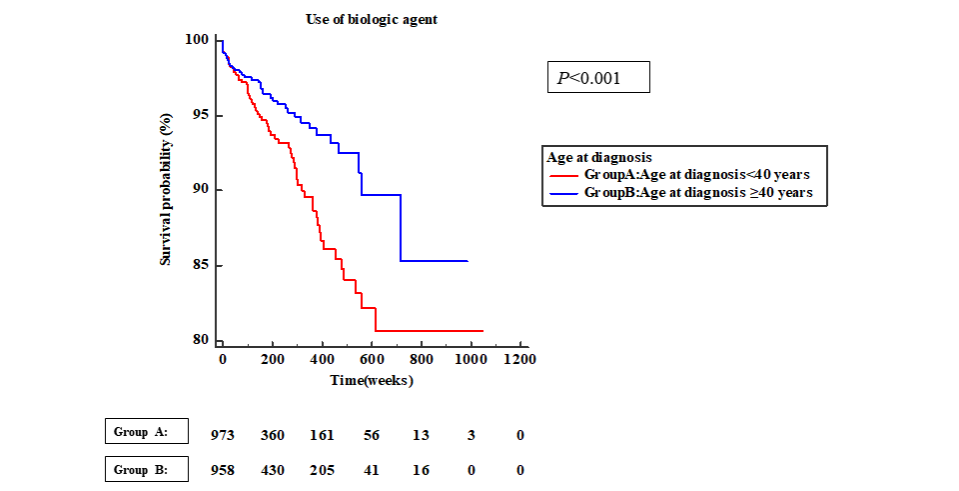
Kaplan-Meier analysis for experience of biologic agents in patients with ulcerative colitis.

**Figure 3:**
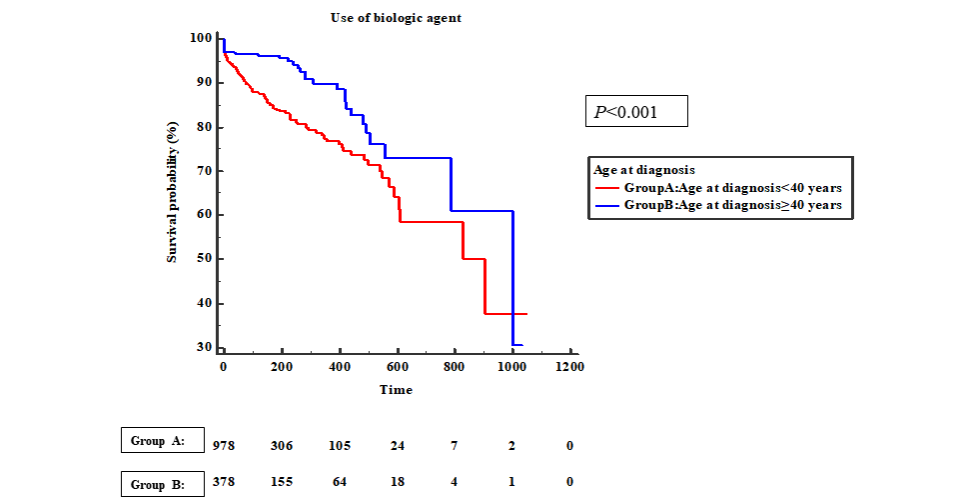
Kaplan-Meier analysis for experience of biologic agents in patients with Crohn's disease.

The EO UC group had a significantly lower event-free survival rate than that of the LO UC group (*P*<.001). The rate of biologic-agent therapy initiation was significantly higher in the EO UC group than in the LO UC group (*P*<.001) (Figure 2). The rate of biologic-agent initiation therapy was also significantly higher in the EO CD group than in the LO CD group (*P*<.001) in the total K-CDM population (Figure 3).

### Factors Related to Previous Biologic-Agent Therapy

The Cox proportional hazards analysis showed that after adjustment for covariates, the odds ratio (OR) for the initiation of biologic-agent therapy in the EO UC group compared with the LO UC group was 2.3 (95% CI 1.3-3.8, *P*=.002) (Table 4). The OR for initiation of biologic-agent therapy in the EO CD group compared with the LO CD group was 5.4 (95% CI 1.9-14.9, *P*=.001) (Table 5).

**Table 4:** Multivariate analysis for the detection of associative valuables with experience of biologic agent in ulcerative colitis.

Characteristics		Odds ratio (95% CI)	*P* value
**Sex**			
	Male	1.4 (0.8-2.3)	.19
**Age at diagnosis**			
	<40 years	2.3 (1.3-3.8)	.002
**Phenotype of inflammatory bowel disease**			
	Systemic steroid uses at the diagnosis	2.1 (1.2-3.6)	.01
**Laboratory findings**			
	Hemoglobin <10 g/dL	1.1 (0.5-2.2)	.79
	C-reactive protein ≥3 g/dL	1.9 (1.1-3.4)	.02
	Albumin <3.5 g/dL	2.2 (1.2-3.9)	.01

**Table 5:** Multivariate analysis for the detection of associative valuables with experience of biologic agent in Crohn's disease.

Characteristics		Odds ratio (95% CI)	*P* value
**Sex**			
	Male	0.9 (0.5-1.7)	.81
**Age at diagnosis**			
	<40 years	5.4 (1.9-14.9)	.001
**Phenotype of inflammatory bowel disease**			
	Systemic steroid uses at the diagnosis	2.2 (1.2-4.1)	.009
**Laboratory findings**			
	Hemoglobin <10 g/dL	1.4 (0.7-2.9)	.31
	C-reactive protein ≥3 g/dL	1.7 (0.9-2.9)	.05
	Albumin <3.5 g/dL	1.5 (0.8-2.8)	.19
	High-density lipoprotein cholesterol ≤40 g/dL	1.2 (0.7-2.0)	.49

## Discussion

### Principal Results

In this study we found that patients with EO IBD had a worsened prognosis in terms of the first administration of biologic agents than patients with LO IBD. In the Cox proportional hazards analysis, the OR for the initiation of therapy with biologic agents was 2.3 (95% CI 1.3-3.8, *P*=.002) in the EO UC group compared with the LO UC group. For CD, the OR was 5.4 (95% CI 1.9-14.9, *P*=.001) in the EO CD group compared with the LO CD group.

We also validated the utility of the K-CDM model for multicenter gastrointestinal studies in terms of its accuracy, efficacy, and transparency. To our knowledge, this is the first study to apply and validate the CDM for gastrointestinal research. We first transformed the EMRs to the K-CDM version 5.0 and subsequently assessed the association of the age at diagnosis with the prognosis of IBD using the K-CDM network data.

### Comparison With Prior Work

The K-CDM uses the OHDSI database system, which aims to facilitate global, large-scale observational research that is reproducible, because it is based on CDMs and queries [18,36-39]. CDMs were developed to enable management of large amounts of data in the medical field. The use of standardized CDMs in research has several advantages, including speed and the use of standard analytical tools for different EMR database systems [18,38-44]. In this study, we used MS-SQL (Microsoft, Redman, WA) data-management software to analyze the EMR data from several tertiary medical centers.

There have been several attempts to use CDMs in the medical field [4,45-48]. Yue et al [49] used CDMs in studies on traumatic brain injury and overviewed the pertinent traumatic brain injury modules and CDMs. Amel et al [50] evaluated the clinical outcomes of mitochondrion-related diseases using a CDM specific to neurological diseases. Panaccio et al [51] used a CDM to analyze the hospitalization and mortality rates of patients with atrial fibrillation using a standardized methodology as well as coding algorithms across two types of data sources. However, no gastrointestinal study to date has used a CDM. In this study, we validated the utility of a CDM for gastrointestinal research.

Unlike other disease-specific CDMs [46,51], the K-CDM transforms almost all of the outpatient and inpatient data in each hospital. Therefore, the K-CDM data can be used for research related to a variety of medical specialties [16-18]. Moreover, the K-CDM is based on the OHDSI database framework, which enables its use in multicenter studies worldwide.

In this study, we found that age at diagnosis was associated with a poor prognosis of IBD (ie, use of biologic agents) [10,11,15,52], and that EO UC and EO CD were associated with more frequent exacerbation events and earlier initiation of therapy with a biologic agent. Balde et al [53] reported that the use of biologic agents was more frequent in French patients with EO CD, which suggests a poor prognosis. Hwang et al [35] reported that among 1382 Korean patients with CD, the EO group had a worse prognosis, as reflected by a lower frequency of biologic agent use during the follow-up period.

In Korea, there have been emerging movements to share EMR data in the form of CDMs. To achieve this data-sharing process, more than 40 tertiary medical centers in Korea have made efforts to transform their EMR data in to CDM format using OHDSI open-source resources since 2018. Before the launching of the formal OHDSI platform-based study, we used Atlas or Achilles tools to build codes and extract data from the individual institutes and then analyzed the results in a meta-analysis to protect the distributed data system concepts; we intended to determine if gastroenterology researches using CDMs were more accurate and convenient than conventional study processes. We extracted the CDM-based data from the GMC and K-CDM network using MS-SQL and merged the data for further logrank tests and Cox proportional analyses. Even though this was not identical to typical OHDSI network studies, our study process had value by validating the CDM model in gastrointestinal research.

### Limitations

Studies using the K-CDM have several limitations. First, many IBD-related factors, including disease activity at the time of diagnosis, initial UC Mayo score, and the CD activity index, were not included. Instead, we regarded use of systemic steroids or other immunosuppressive agents at the time of diagnosis as indicative of disease activity. The Korean IBD treatment guidelines state that systemic steroids or other immunosuppressive agents should be prescribed only to patients with moderate or severe diseases [33]. Moreover, in Korea, biologic-agent therapy is typically delayed until the second exacerbation event or until the patient is unresponsive to, or dependent on steroids or immunosuppressants for at least 3 months after the diagnosis of IBD. Therefore, in Korea, the use of biologic agents is indicative of a poor prognosis [33]. The UC Mayo score and CD activity index reflect the disease severity. Systemic steroid use at the time of diagnosis is indicative of moderate-to-severe and severe IBD activities. Thus, we used the systemic steroid use at the time of diagnosis as the operational definition of the UC Mayo score and the CD activity score. Gastroenterologists should focus on and make efforts to qualify the variables in the K-CDM network in gastrointestinal research. It is promising that the majority of the clinical contents used in gastrointestinal research could be equipped in the K-CDM tables, especially through the standardized clinical data domain, once researchers qualify the variables of the K-CDM. Second, this was a retrospective study and thus may have been influenced by selection or indication bias. Third, inclusion of only tertiary medical centers may have introduced selection bias.

### Conclusion

In conclusion, patients with EO IBD have a worse prognosis than patients with LO IBD. Moreover, we successfully validated that the K-CDM network database enables physicians to conduct multicenter gastroenterology studies with more efficient and transparent study processes.

## Abbreviations

CD: Crohn’s disease
CDM: common data model
EMR: electronic medical record
EO: early-onset
GMC: Gill Medical Center
IBD: inflammatory bowel disease
K: Korean
LO: late-onset
OHDSI: Observational Health Data Sciences and Informatics
OR: odds ratio
UC: ulcerative colitis
